# Rs2459976 in *ZW10* gene associated with congenital heart diseases in Chinese Han population

**DOI:** 10.18632/oncotarget.23240

**Published:** 2017-12-13

**Authors:** Chao-Yu Sun, Chi Sun, Rui Cheng, Shuai Shi, Ying Han, Xue-Qi Li, Ji-Xin Zhi, Fei-Feng Li, Shu-Lin Liu

**Affiliations:** ^1^ Systemomics Center, College of Pharmacy and Genomics Research Center, State-Province Key Laboratory of Biopharmaceutical Engineering, Harbin Medical University, Harbin, China; ^2^ Department of Cardiology, Fourth Affiliated Hospital, Harbin Medical University, Harbin, China; ^3^ Translational Medicine Research and Cooperation Center of Northern China, Heilongjiang Academy of Medical Sciences, Heilongjiang, China; ^4^ Department of Microbiology, Immunology and Infectious Diseases, University of Calgary, Calgary, Canada

**Keywords:** congenital heart disease, ZW10, human embryonic stem cells, SNP, ASD

## Abstract

Congenital heart diseases (CHD) are a large group of prevalent and complex anatomic malformations of the heart, with the genetic basis remaining largely unknown. Since genes or factors associated with the differentiation of human embryonic stem (HES) cells would affect the development of all embryonic tissues, including cardiac progenitor cells, we postulated their potential roles in CHD. In this study, we focused on ZW10, a kinetochore protein involved in the process of proper chromosome segregation, and conducted comparative studies between CHD patients and normal controls matched in gender and age in Chinese Han populations. We identified three variations in the *ZW10* gene, including rs2885987, rs2271261 and rs2459976, which all had high genetic heterozygosity. Association analysis of these genetic variations with CHD showed correlation between rs2459976 and the risk of CHD. We conclude that rs2459976 in the *ZW10* gene is associated with CHD in Chinese Han populations.

## INTRODUCTION

Congenital heart diseases (CHDs) are a large group of prevalent and complex anatomic malformations of the heart, with the incidence of about 7.5% in newborns [1], and about 1% of the CHD patients would require clinical intervention [2]. The most common types of CHDs include tetralogy of Fallot, ventricular septal defects, pulmonary stenosis, patent ductusar teriosus, and mitral valve insufficiency [3, 4]. CHDs are often complicated with malignant arrhythmias or heart failure, which may lead to death [5]. Although many chromosomal variants have been identified for their associations with the illness [6–8], much remains unknown about the relationships between the genetic abnormalities and CHDs.

The mammalian heart is one of the first formed organs during embryogenesis. In the process, the human embryonic stem (HES) cells differentiate to various cell types of the ectoderm, endoderm and mesoderm, and cardiomyocytes are generated in the mesoderm [9]. The mammalian heart consists of several cell types [10], including myocardium cells, cardiac neural crest (NC) cells, aorticopulmonary septum cells and membranous ventricular septum cells [11]. These cardiac progenitors interact to form the heart and many gene defects or variants in the cells have been reported for their relevance with the heart malformations, such as muscle segment homeobox 1 (MSX1) [1, 12, 13]. In a previous study, we found that variants in the *MSX1* gene are closely associated with the risk of ventricular septal defect (VSD, a common type of CHD) [14]. Additionally, our recent work has also demonstrated the associations of variants in other genes with the risk of CHD, such as those of the *STX18* gene [15]. STX18 is involved in several cell processes, such as transporting vesicle membrane fusion with target compartments in the cellular activities [16] and forming complexes with cell cycle-related proteins [17, 18]. These findings suggest that genes associated with cellular genesis processes may affect the development of all embryonic tissues, including cardiac progenitor cells and therefore might be associated with the pathogenesis of CHDs. For example, the kinetochore protein ZW10 plays key roles in the cell cycle, especially at the cell division stage, so we postulated the potential involvement of ZW10 in CHD.

ZW10 ensures proper chromosome segregation and participates in turning off spindle checkpoint activity, preventing cells from prematurely exiting mitosis [19–22]. When inactive ZW10 and colchicine exist, the cells of *Drosophila melanogaster* showed chromosome missegregation and abnormal separation of sisterchromatids [20]. Similar phenomena were observed in HeLa cells when anti-ZW10 antibody was applied [21, 19, 23, 24]. Based on the documented key roles of ZW10 in the cellular processes, we postulate its association with CHD. To validate this postulation, we compared the gene sequences between 300 Chinese Han CHD patients and 600 controls and found that the variation rs2459976 in the *ZW10* gene was associated with the risk of CHD.

## RESULTS

### Patients

Clinical diagnosis of all the participants was confirmed at the Second or the Fourth Affiliated Hospital of Harbin Medical University. The participants had no history or manifestations of any other systemic abnormalities. We also established that their mothers did not take medicines or attract infections during gestation, because these factors had been found to be associated with heart malformation in pregnancy [25, 26].

A total of 300 CHD patients and 600 unrelated controls were recruited for this study, and there were no statistical differences in age or gender composition between the two groups (Table 1). The 300 CHD patients contained 122 with ventricular septal defects (VSD), 109 with atrial septal defects (ASD), 46 with patent ductus arteriosus (PDA),11 with tetralogy of Fallot, and 12 with other types of congenital heart defects.

**Table 1:** General information of the study population

Parameter	CHD	Control	*F*	*t*	*P*	95% CI-Up	95% CI-Low
***Sample (n)***	300	600	-	-	-	-	-
***Male/Female (n)***	134/166	260/340	-	-	0.704	-	-
**Age (years)**	15.42 ± 17.00	14.02 ± 9.31	185.530	1.581	0.114	−0.33561	3.11994

Data are shown as mean ± SD; between the two groups, there were no statistical differences of the age and gender composition.

### *ZW10* gene analysis

**Figure 1:**
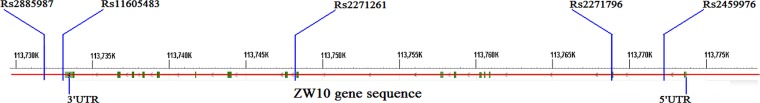
Schematic diagramoflocations of ZW10 gene SNPsrs2885987, rs11605483, rs2271261, rs2271796 and rs2459976

We sequenced the *ZW10* gene to test the hypothesis that germline common variants in *ZW10* may confer susceptibility to CHD. We identified rs2885987 and rs11605483 near the 3′UTR, rs2271261 and rs2271796 within the intron region, and rs2459976 near the 5′UTR of the gene (Figure 1). The genetic heterozygosity of the rs11605483(T/T, 0.95; C/T, 0.05) and rs2271796 (T/T, 0.99; C/T, 0.01) loci was very low. On the other hand, the genetic heterozygosity of rs2885987,rs2271261 and rs2459976 in the gene *ZW10* was considerably high, so we focused on the latter three variants for further analysis.

### Statistical analysis of SNPs rs2885987, rs2271261 and rs2459976

We conducted analyses on the three SNPs and found that rs2459976 was associated with the risk of CHD in the Chinese Han population (Chi-square value: 7.153; *P* value: 0.028), but rs2885987 and rs2271261 were not (Tables 2 and 3). The Hardy-Weinberg equilibrium test for the CHD and controls were conducted and it was in line with the equilibrium (Table 4).

**Table 2:** The genotypes and allele frequencies of SNPs rs2459976, rs2271261 and rs2885987 in 300 Chinese Han CHD patients and 600 non-CHD controls

SNP	Group		Genotype frequency (%)			Allele frequency (%)	
***rs2459976***	**Genotype**		**C/C**	**C/T**	**T/T**	**C**	**T**
	CHD	300	167 (55.67)	108 (36.00)	25 (8.33)	442 (73.67)	158 (26.33)
	ASD	109	57 (52.29)	39 (35.78)	13 (11.93)	153 (70.18)	65 (29.82)
	VSD	122	67 (54.92)	47 (38.52)	8 (6.56)	181 (74.18)	63 (25.82)
	Controls	600	281 (46.83)	271 (45.17)	48 (8.00)	833 (69.42)	367 (30.58)
***rs2271261***	**Genotype**		**T/T**	**T/A**	**A/A**	**T**	**A**
	CHD	300	223 (74.33)	69 (23.00)	8 (2.67)	515 (85.83)	85 (14.17)
	ASD	109	73 (66.97)	35 (32.11)	1 (0.92)	181 (83.03)	37 (16.97)
	VSD	122	88 (72.13)	29 (23.78)	5 (4.10)	205 (84.02)	39 (15.98)
	Controls	600	460 (76.67)	133 (22.17)	7 (1.17)	1053 (87.75)	147 (12.25)
***rs2885987***	**Genotype**		**T/T**	**T/G**	**G/G**	**T**	**G**
	CHD	300	230 (76.67)	67 (22.33)	3 (1.00)	527 (87.83)	73 (12.17)
	ASD	109	80 (73.39)	28 (25.69)	1 (0.92)	188 (86.24)	30 (13.76)
	VSD	122	99 (81.15)	21 (17.21)	2 (1.64)	219 (89.75)	25 (10.25)
	Controls	600	478 (79.67)	111 (18.50)	11 (1.83)	1067 (88.92)	133 (11.08)

**Table 3:** Associations of rs2459976, rs2271261 and rs2885987 variants in *ZW10* with risk of CHD in Chinese Han populations

Title			Pearson Chi-square				Risk		
Genotyped SNP	Disease Type	Statistical Types	Value	Min expect count	df	Asymp. Sig. (2-sided)	OR	95% CI-Up	95% CI-Low
***rs2459976***	CHD-Control	Genotype	7.153	24.33	2	0.028^*^	--	--	--
		Allele	3.497	175.0	1	0.061	0.811	0.652	1.010
	ASD-Control	Genotype	4.089	9.38	2	0.129	--	--	--
		Allele	0.51	66.41	1	0.821	0.964	0.704	1.321
	VSD-Control	Genotype	2.662	9.46	2	0.264	--	--	--
		Allele	2.200	72.66	1	0.138	0.790	0.578	1.079
***rs2271261***	CHD-Control	Genotype	2.905	5.00	2	0.234	--	--	--
		Allele	1.309	77.33	1	0.253	1.182	0.887	1.575
	ASD-Control	Genotype	5.053	1.23	2	0.080	--	--	--
		Allele	3.644	28.29	1	0. 056	1.464	0.988	2.170
	VSD-Control	Genotype	5.634	2.03	2	0.060	--	--	--
		Allele	2.519	31.43	1	0.112	1.363	0.929	2.000
***rs2885987***	CHD-Control	Genotype	2.608	4.67	2	0.271	--	--	--
		Allele	0.463	68.67	1	0.496	1.111	1.820	1.506
	ASD-Control	Genotype	3.349	1.84	2	0.187	--	--	--
		Allele	1.301	25.06	1	0.254	1.280	0.837	1.959
	VSD-Control	Genotype	0.141	2.20	2	0.932	--	--	--
		Allele	0.146	26.70	1	0.702	0.916	0.583	1.438

^*^:Statistically significant

**Table 4:** The CHD and controls groups were in line with Hardy-Weinberg equilibrium

Group		Genotype	H-W equilibrium Testing		
SNP	Test	Homo/Hetero/Homozygote	0 (HET)	E (HET)	*P*
***rs2459976***	All	73/379/448	0.4211	0.4132	0.6283
	Affected	25/108/167	0.3600	0.3880	0.2331
	Unaffected	48/271/281	0.4517	0.4246	0.1488
***rs2271261***	All	15/202/683	0.2244	0.2246	1.0000
	Affected	8/69/223	0.2300	0.2432	0.3417
	Unaffected	7/133/460	0.2217	0.2150	0.5694
***rs2885987***	All	14/178/708	0.1978	0.2027	0.5087
	Affected	3/67/230	0.2233	0.2137	0.5927
	Unaffected	11/111/478	0.1850	0.1971	0.1439

**Figure 2:**
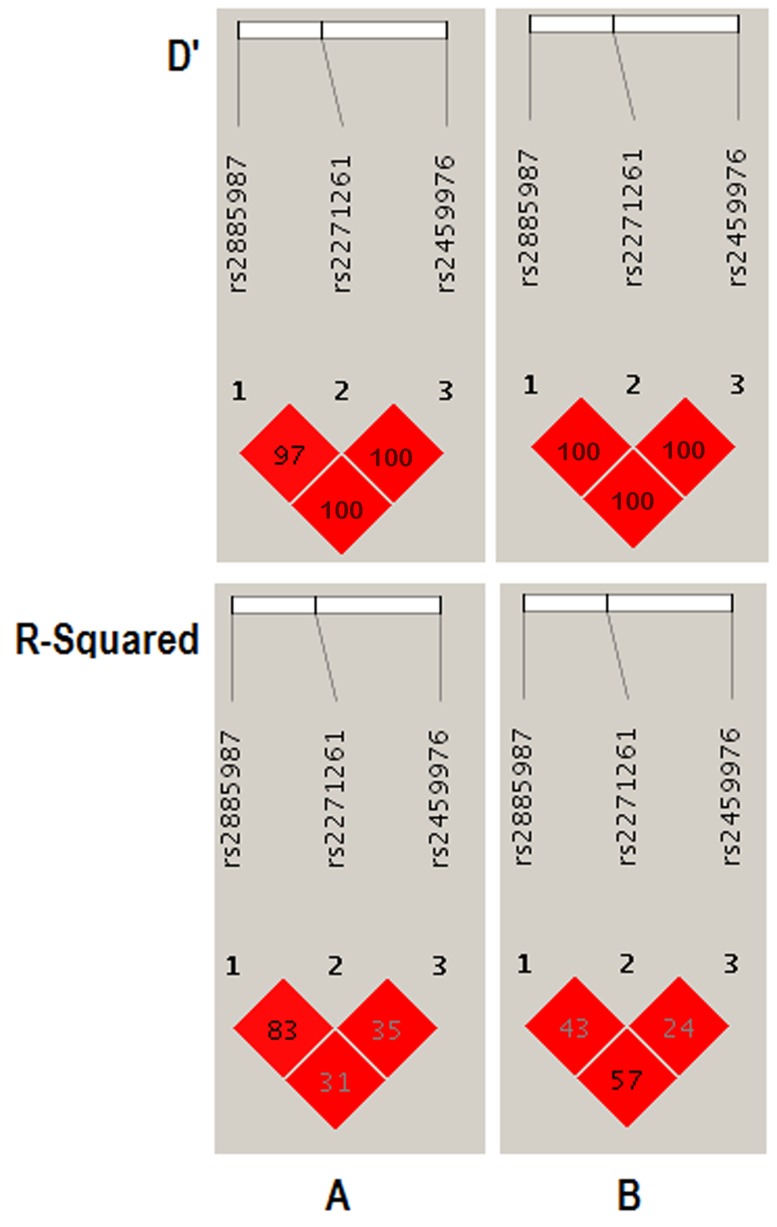
LD analysis of the variants rs2885987, rs2271261 and rs2459976 in the *ZW10* gene The LD plots were generated using the Haploview software v4.2. (**A**) Data analysis between CHD patients and controls from the present study of variants in *ZW10* gene; (**B**) Data from HapMap CHB of variants in *ZW10* gene. The data from the HapMap CHB and this work were consistent.

The experiment-wide significance threshold of the variant rs2459976 in *ZW10* gene was 0.026. The Haploview software was used to conduct LD analysis of the variants rs2885987, rs2271261 and rs2459976, and the results were consistent with the data from the HapMap CHB population (Figure 2). The genotype frequencies in the CHD and control groups were further analyzed by three genetic models, including trend, dominant and recessive models, in addition to chi-square and fisher tests (Table 5). All results indicated that the variant rs2459976 was associated with the risk of CHD.

**Table 5:** SNP rs2459976 within ZW10 gene associated with the risk of congenital heart diseases in Chinese Han populations

Disease Type	Value	Trend model	Dominant model	Recessive model
CHD-Control	ChisQ	3.565	6.242	0.030
	*P*	0.0590	0.0125^*^	0.8629
ASD-Control	ChisQ	0.053	1.102	1.809
	*P*	0.8181	0.2937	0.1787
VSD-Control	ChisQ	2.324	2.654	0.295
	*P*	0.1274	0.1033	0.5871

^*^:Statistically significant.

## DISCUSSION

In this study, we analyzed the transcribed regions and splicing sites of the kinetochore protein gene *ZW10* in a large cohort of CHD patients and controls, finding that the variant rs2459976 in the *ZW10* gene was associated with the risk of CHD in the Chinese Han population. Further work is needed to determine whether the kinetochore protein ZW10 may be involved in the CHD etiology.

The mammalian heart is a complex organ and its formation involves many genes with strict temporal, spatial and sequential expression [3]. Additionally, there are several cell types with distinct lineage origins in the mammalian heart [10, 11], interactions of which are necessary in the process of the cardiac development [1]. In previous studies, we found that variants in the *STX18* and *MSX1* genes are associated with CHDs, probably due to the regulating effects of their protein products on cell cycle [14, 15]. STX18 also transports proteins between the cellular endoplasmic reticulum (ER) and Golgi [27]. On the other hand, *MSX1* is an active gene in the HES cells: *MSX1* is up-expressed when the HES cells are co-cultured with PA6 cells [28] and is lower expressed when the HES cells are treated with dopamine [29]. In the embryonic development, HES cells differentiate to various cell types including cardiomyocytes in the mesoderm [9]. These findings suggest that any defects in highly expressed genes or factors in embryonic cells, such as HES cardiac progenitor cells, may affect the development of the heart.

The kinetochore protein ZW10 is expressed consistently through out the cell cycle in the cardiac progenitor cells during embryonic development [30]. It co-localizes with the ER, participates in the ER and Golgi trafficking, takes part in the mitotic checkpoint and interacts with the dynactin subunit dynamitin [20, 24]. Additionally, ZW10 associates with specific interphase structures and regulates transport movement of Golgi, endosome, and lysosome [31]. In the present work, we found that the variant rs2459976 in the *ZW10* gene was associated with the risk of CHD.

Inmembrane budding and fusion, the N-terminal fragment of ZW10 interacts with STX18 and participates in STX18 mediated membrane sorting [24].The C-terminal end of ZW10 contains part of the dynamitin-interacting region, by which ZW10 anchors dynein to membranous organelles [31]. Whereas the C-terminal region of ZW10 exhibits weak interactions with dynamitin [32], the N-terminal region of ZW10 interacts with dynamitinin in amutually exclusive manner [32, 33]. The variant rs2459976 is located within the region between exons 1 and 2 of the ZW10 gene, which encodes the N-terminal region of ZW10 protein. The locations of the variants rs2271261 and rs2885987 are far from the N-terminal region of ZW10 protein, and we did not find any statistical significance between the two variants and CHD.

The 3′UTR and 5′UTR regions contain functional sequences that regulate the expression of many genes [34, 35] and may be associated with disease pathogenesis [36–39]. In a previous study, we identified variants near or within the 3′UTR or 5′UTR of genes that are associated with the risk of CHD [14, 40]. All these previous findings demonstrate the important roles of 5′UTR and 3′UTR on the functions of genes and their involvement in diseases. Although results in the current study support the possible involvement of the kinetochore protein gene *ZW10* in the etiology of CHD, further validation is necessary to distinguish its roles in the CHD pathogenesis from associations just as a by stander.

In conclusion, we compared the *ZW10* gene sequences between 300 Chinese Han CHD patients and 600 controls, and revealed a correlation between rs2459976 and the risk of CHD in the Chinese Han population.

## MATERIALS AND METHODS

### The study populations

We gathered 300 CHD patients and 600 normal controls for this study, from the Second and the Fourth Affiliated Hospitals of Harbin Medical University, Harbin, China; specimens of 260 of the 300 CHD patients were overlapping with those used for another study [3, 40]. Although we collected the cohort from two hospitals, the patients and controls were ethnically matched and their relatives were excluded from this study. All the CHD patients and normal controls were given comprehensive physical, electrocardiogram and ultrasonic echocardiogram examinations. None of the patients showed any other cardiac or systemic abnormalities, and the normal controls did not show any defects in the heart or other body parts. For this work, we obtained a written informed consent from each participant or their parents on behalf of minors, and the Ethics Committee of the Harbin Medical University approved this work. All procedures performed in studies involving human participants were in accordance with the ethical standards of the institutional and/or national research committee and with the 1964 Helsinki declaration and its later amendments or comparable ethical standards.

### DNA analysis

Genomic DNA was extracted from the peripheral blood leukocytes of the participants as detailed in previous studies [15, 41]. The human *ZW10* gene is located on 11q23.2. To determine the SNP genotypes in the gene, we amplified the exons and splicing sites, 3′UTR and 5′UTR of the *ZW10* gene using the polymerase chain reaction (PCR) method (Supplementary Table 1), and sequenced the products by the Sanger technique using standard protocols [42]. After that, the genotypes were determined using PCR and gene sequencing methods [3, 43].

### SNP genotyping analysis

We determined genotypes of rs2885987, rs11605483, rs2271261, rs2271796 and rs2459976 in the *ZW10* gene (Figure 1) on 300 CHD patients and 600 normal controls, and two independent researchers conducted the measurements. The overall CHD statistical analysis was conducted according to the types of CHD and sample sizes.

### Statistical analysis

The statistical analyses and Hardy-Weinberg equilibrium tests of the CHD and control populations were conducted by the software and methods as previously reported [15]. The experiment-wide significance threshold, matrix of linkage-disequilibrium (LD) correlation for the markers and haplotype diagram of LD structure were calculated using the online software SNPSpD (http://neurogenetics.qimrberghofer.edu.au/SNPSpD/) [44] and Haploview software (http://www.broadinstitute.org/scientific-community/science/programs/medical-and-population-genetics/haploview/haploview) [45], respectively.

### Patient consent

Informed consent was obtained from all individual participants included in the study.

### Ethics approval

This work has been approved by the Ethics Committee of Harbin Medical University.

## SUPPLEMENTARY MATERIALS TABLE



## Abbreviations

CHD: Congenital heart disease
ASD: atrial septal defect
VSD: ventricular septal defect
HES: human embryonic stem
LD: linkage-disequilibrium
