# Supplementary material for: Rs2459976 in ZW10 gene associated with congenital heart diseases in Chinese Han population
Source: Oncotarget. 2017 Dec 13;9(3):3867–74. doi: 10.18632/oncotarget.23240 (PMC5790506; doi:10.18632/oncotarget.23240)
Supplement: Supplementary file 1 [file oncotarget-09-3867-s001.pdf]

Rs2459976 in *ZW10* gene associated with congenital heart diseases in Chinese Han population

SUPPLEMENTARY MATERIALS

Supplementary Table 1: PCR primers used for *ZW10* gene sequence analysis

Exon	Forward primer	Reverse primer	Size (bp)	Tm (°C)
1	CACCGTGGAAATCCCATCT	CACTCTGTCTGCCCTTAGCC	398	58.7
2	TGCCTTGTGGAAGATGAA	CCACCTCCCTACAAACCT	262	49.6
3	AGGATAAGGGTTTGGACT	TTAAGAAATGCTCCCACT	462	47.2
4	GGAGGGAAAGTGGGAGCA	ATCTGGACTCAGGGCAAT	276	47.9
5	TTCTAATTGCCCTGAGTC	CAGATGAGTTGGTCCTAA	384	47.2
6	GTGCTGGGATTACAGACA	TACTGGGCCTCAGGAGAT	426	48.4
7	CCAAAGTGCTGGGATTAC	CTGATGGCCCTACTTGTC	496	48.5
8	TCCCACGCAGTTGAGAAG	GTAGGAAGCCAGGAGAAA	475	47.8
9	AATTTAAGGCTGGAAATATG	CAGCTCAACAACCGTCAT	452	46.9
10	CTGACCTCGTGATCTGCC	GATATGAAAACCCAAAGAA	480	48.6
11	GGTGTTCTGTGCAACTGT	GTAATAAATGACCAAATCCT	368	44.9
12	ATTGTTAATCACCAGGATAG	GATACAGCTCAGGACCAC	507	49.2
13	GGCAGAACAGATACATGG	GGGGTGCAGGGAATCAAC	351	48.1
14	AGGTTGATGAGTCTGCTT	GGTAGGCTGAGAAATAGT	540	46.8
15	CTGCCCTTTCCCCACTTC	CCCCAGCTACTGCTACTCAA	406	51.4
16a	GGAACTCTATCTTTCGTTT	CCTGACTCCTGGAATCTC	512	49.2
16b	TGTCAAGTAAAGCACCTC	AAGGATGGCATAGAATGT	392	48.0
